# The role of telemedicine in the context of the COVID-19 pandemic in Colombia

**DOI:** 10.3389/fpubh.2025.1667349

**Published:** 2025-11-18

**Authors:** Susana Orrego Villegas, Eric Adjei Boakye, Jafet Arrieta, Karine Posada España, Melisa Naranjo Vanegas, Gloria Prado Pino

**Affiliations:** 1 Harvard Medical School, Boston, MA, United States; 2 Universidad CES, Medellín, Colombia; 3 Seguros SURA, Medellín, Colombia; 4 Department of Public Health Sciences, Henry Ford Health, Detroit, MI, United States; 5 Institute for Healthcare Improvement, Boston, MA, United States; 6 Harvard TH Chan School of Public Health, Boston, MA, United States; 7 Bioscience Center – Ayudas Diagnósticas Sura, Medellín, Colombia; 8 Universidad Tecnológica del Chocó Diego Luis Córdoba UTCH, Quibdó, Colombia; 9 Organización Panamericana de la Salud (PAHO), Washington, DC, United States

**Keywords:** telemedicine, COVID-19 pandemic, healthcare access, Colombia, digital health equity

## Abstract

**Background:**

With the onset of the COVID-19 pandemic in Colombia, telemedicine (TM) became a key strategy to sustain healthcare delivery for both acute and chronic conditions. This study aimed to describe the utilization and characteristics of TM during the pandemic in Colombia.

**Methods:**

We conducted a retrospective, descriptive study using administrative and clinical records from a major Colombian health insurance provider covering approximately 15% of the national population. We analyzed all TM consultations between March 2020 and June 2021, assessing sociodemographic, geographic, and clinical characteristics. TM users and the overall insured population were described, and primary care unit (PCU) locations were georeferenced.

**Results:**

A total of 10,016,931 individuals were enrolled in the health insurance plan, of whom 2,633,564 used TM services during the study period. TM users were predominantly female (62.8%) and aged 19–45 years (55.3%). Most belonged to low-income groups (64.9%) and were affiliated with the subsidized income level group. The most frequent diagnosis was COVID-19 (28.3%), followed by general symptoms and unspecified conditions (19.4%). Telemedicine was used nationwide, with higher concentrations in central and northern departments such as Antioquia and Bogotá.

**Conclusion:**

During the pandemic, telemedicine was widely adopted across Colombia and reached large segments of low-income populations, suggesting its potential to support healthcare access in diverse settings. While our descriptive design does not allow for causal claims about effectiveness or equity, these findings highlight the value of TM as a complementary healthcare delivery model. Continued investment in digital infrastructure, workforce training, and primary care networks will be essential for its long-term integration into Colombia’s health system.

## 1 Introduction

Before the COVID-19 pandemic, telemedicine (TM) in Colombia was underutilized and not fully integrated into the healthcare system. Although the Ministry of Health and Social Protection (MHSP) enacted regulations between 2007 and 2019 to promote and standardize TM (1, 2), its implementation remained limited to a few public and private health insurances providers (3, 4). The onset of the pandemic and the nationwide mandatory quarantine from March 24 to July 15, 2020, radically altered healthcare delivery. Routine outpatient visits and elective procedures were suspended, and the healthcare system faced the urgent need to offer medical services virtually (5–7). In response, the Colombian government encouraged both public and private insurers to rapidly expand telemedicine services. As a result, over 3 million TM consultations were conducted in the first 2 months of the pandemic alone (7), and more than 42 million by January 2021 (8–10).

SURA, one of the largest insurers in the country, provided 2.6 million telemedicine consultations between March 2020 and June 2021. SURA covers approximately 30% of the insured population in Colombia, reaching around 13 million individuals across urban and rural regions, with benefit package and copayment structure comparable to those of other major insurers. The broad and diverse coverage reduces the risk of strong selection bias and allows findings from SURA to be more generalizable both within Colombia and to other middle-income countries with similar mixed health systems (11). Despite this massive expansion, there is limited evidence on the profile and distribution of TM users during this period. This knowledge gap has important implications: without a clear understanding of who accessed telemedicine services, policies and programs risk reinforcing inequities in access to healthcare. Evidence from other Latin American countries illustrates these consequences. In Brazil, targeted investments in digital health infrastructure during the pandemic improvement continuity of care in underserved areas, reducing disparities in service use (12, 13). By contrast, in Peru, limited adoption and lack of equitable access to TM left vulnerable populations further marginalized during the health emergency.

This study aims to describe the sociodemographic characteristics of TM within a major Colombian insurance provider. The findings may inform future healthcare policies, training and digital health interventions. Moreover, they can provide insights for other health systems in low-and middle-income countries seeking to sustain and scale telemedicine services beyond the pandemic.

## 2 Methods

### 2.1 Study design and population

We conducted a retrospective cohort study of individuals enrolled in the Colombian healthcare insurance provider SURA, which offers coverage under public insurance (EPS SURA), workers´ insurance (ARL SURA), and private insurance plans (Polizas SURA). The study population included all patients who accessed telemedicine (TM) services between March 1, 2020, and June 30, 2021. To contextualized characteristics, we include all individuals enrolled in SURA health plans in 2020 and 2022, regardless of telemedicine use.

### 2.2 Setting

Colombia is a middle-income country with Universal Health Coverage (UHC) since 1993. Individuals are required to enroll in an Entidad Promotora de Salud (EPS), and if employed, they are also covered by an occupational risk insurer (ARL). Additionally, individuals may purchase private insurance plans (Polizas) for extended benefits. Each patient is assigned to a Primary Care Unit (PCU), referred to as IPS (Institución Prestadora de Salud). SURA is one of the main insurers in Colombia, offering all three types of coverage.

### 2.3 Telemedicine access channels

During the study period, SURA offered telemedicine services through four distinct access channels designed to accommodate varying levels of digital literacy and connectivity. These included a mobile application, a toll-free telephone line, a dedicated website, and WhatsApp messaging. This multichannel strategy was implemented to enhance accessibility and ensure broad coverage among diverse population groups.

### 2.4 Data sources and georeferencing

Sociodemographic and clinical data were obtained from SURA electronic health records (EHRs), which encompass all patient interactions across insurance types (EPS, ARL and Polizas). For geospatial analysis, we georeferenced each patient’s assigned Primary Care Unit (PCU) rather than relying on self-reported locations and the time of telemedicine use. The geographic coordinates (latitude and longitude) of each PCU were extracted from the administrative registry and used to generate geographic information system (GIS) heat maps that visualized the spatial distribution and intensity of telemedicine utilization across the country.

### 2.5 Variables and measures

We analyzed several sociodemographic and clinical variables. Age was categorized into five groups: children (0–10 years), adolescents (11–18 years), young adults (19–45 years), middle-aged adults (46–60 years), older adults (61–70 years), and older adults (>70 years). Educational attainment was classified into five categories: primary education, secondary/high school, occupational/technical degree (e.g., electrician, culinary training), graduated education (specialization, master’s, PhD, postdoctoral), and unknown or not reported. Income level was determined based on the 2023 Colombian Minimum Monthly Wage (MMW), defined as 1,160,000 Colombian pesos (approximately 240.5 USD at an exchange rate of 1 USD = 4,824.25 COP on March 21, 2023). Income categories included: subsidized (unemployed), low income (1–2 × MMW), middle income (3–5 × MMW), and high income (>5 × MMW). Geographically, patients were grouped into five microregions based on administrative divisions and patterns of healthcare organization: Antioquia, Center-East-South, Eje Cafetero, North, and West. Ethnicity was recorded using standard Latin American classifications, including Black, White, Indigenous, Mestizo (Indigenous and European ancestry), Mulato (African and European ancestry), and Zambo (African and Indigenous ancestry). Additionally, diagnoses made during telemedicine encounters were extracted from EHRs using ICD-10 codes to assess the distribution of health conditions addressed through TM.

### 2.6 Statistical analysis

Descriptive statistics (frequencies and proportions) were computed for all variables of interest. For spatial analysis, we used ArcGIS Pro software to generate density-based heat maps of telemedicine utilization. Locations with the highest concentration of TM users—defined as clusters with over 100,000 consultations—were highlighted using red markers. All statistical analyses were conducted using StataBE 17 and Microsoft Excel.

## 3 Results

### 3.1 Telemedicine use and population characteristics

Between March 2020 and June 2021, a total of 10,016,931 individuals were enrolled in a Colombian health insurance plan; of whom 2,633,564 accessed at least one TM consultation. Among TM users, 94.0% had one appointment, 4.5% had two, and 1.4% had three or more consultations during the study period. Table 1 presents the sociodemographic characteristics of TM users and the overall insured population enrolled between 2020 and 2022. Approximately 63% of the TM users were female, 55.3% were aged 19 to 45 years, 53.1% were assigned to primary care units (PCUs) located in the department of Antioquia, 16.3% reported being mestizo as their ethnicity, 28.6% indicated that their highest educational attainment was a technical or occupational degree, and 64.9% were classified as belonging to a low-income category, defined as earning between one and two Colombian Minimum Monthly Wages (MMW) in 2023. In the entire insured population, 48.4% were female, 51.3% were aged 19 to 45 years, 41.8% were located in the department of Antioquia, 28.9% reported being mestizo as their ethnicity, 20.1% indicated that their highest educational attainment as technical or occupational degree, and 65.5% were classified as being in a low-income category.

**Table 1:** Characteristics of the telemedicine users vs. entire population in health plan.

Characteristics	Telemedicine users *N =* 2,633,564 (%)	Health plan population *N =* 10,016,931 (%)
Female sex – no (%)	1,654.402 (62.8)	4,853,109 (48.4)
Age group – no (%)		
0–10 yr	95,548 (3.6)	628,550 (6.3)
11–18 yr	87,797 (3.3)	659,306 (6.6)
19–45 yr	1,457,548 (55.3)	5,140,508 (51.3)
46–60 yr	505,454 (19.2)	2,486,283 (24.8)
61–70 yr	259,949 (9.9)	598,974 (5.9)
> 70 yr	227,268 (8.6)	503,310 (5.0)
Region– no (%)^		
Antioquia	1,399,316 (53.1)	2,791,999 (41.8)
Center, East, South	513,366 (19.5)	1,773,887 (22.8)
Eje Cafetero	24,367 (1.0)	1,464,189 (14.9)
North	409,347 (15.5)	311,323 (7.2)
West	287,168 (10.9)	1,041,969 (13.3)
Race – no (%)*^^		5,075,259*
Black	33,451 (0.6)	30,769 (1.2)
White	239,020 (4.7)	282,170 (10.7)
Mulatto	5,067 (0.09)	3,619 (0.14)
Indigenous	807 (0.01)	631 (0.01)
Mestizo	829,767 (16.3)	761,877 (28.9)
Zambo	2,430 (0.04)	1,524 (0.06)
Prefered not to answer	3,964,717 (78.0)	1,552,974 (58.9)
Highest Education Achieved- no (%)*		5,075,259*
Primary School	39,837 (1.5)	44,154 (0.9)
High School	129,830 (4.9)	153,272 (3.0)
Occupational degree^a^	755,916 (28.6)	1,024,187 (20.1)
Undergraduate	19,188 (0.7)	19,793 (0.39)
Graduate^b^	7,541 (0.39)	7,105 (0.14)
Prefered not to answer	1,681,252 (63.8)	3,826,748 (74)
Income Level no(%)*^^^		9,017,908**
Subsidized ***	69,502 (2.6)	624,258 (6.9)***
Low income	1,711,007 (64.9)	5,906,638 (65.5)
Middle income	605,574 (22.9)	1,606,370 (17.8)
High income	247,480 (9.4)	880,642 (9.8)

^^^Regions as defined by the health plan. ^*^We received race, Iincome, and highest education data as percentages for September 2022 from the health plan for EPS SURA. To estimate counts for the whole year, we multiplied the percentages by the number of people enrolled for the year (*n =* 5,075,259). ^^^^Races in Latin America: Mestizo: Indigenous + European, Mulato: African + European, Zambo: Black + Indigenous. ^a^Occupations such as electricians and chefs can achieve specific post-high school occupational training. ^b^Graduate programs include: 1-year professional certification, Master’s, PhD and Postdoc. ^**^Income Level: We received percentages for EPS SURA (*n =* 5,075,259) and count data for ARL SURA (*n =* 3,942,649). This information was combined to generate percentages for the general population (*n =* 9,017,908). ^^^^^Categories for income: Subsidized not employ, A or Low income (1- 2X MMW), B or Middle income (3- 5X MMW), C High income (>5X MMW). ^***^Subsidized: This population, is only enrolled in the general insurance (EPS SURA).

### 3.2 Diagnoses and disease burden among telemedicine users

Table 2 details the top ICD-10 diagnoses made during TM consultations. The most frequent clinical diagnosis among TM users was COVID-19 (28.3%). Chronic non-communicable diseases were also common, with hypertension diagnosed in 10.0% and type 2 diabetes mellitus in 3.2% of cases. Acute respiratory infections, mental health disorders, and musculoskeletal complaints were also among the top 10 diagnoses, reflecting a diverse range of clinical needs addressed through TM.

**Table 2:** Principal ICD-10 diagnoses among the telemedicine users.

Number cases	%	Diagnosis
747,462	28.38	COVID-19
512,664	19.47	Z Codes: Factors Influencing Health Status and Contact with Health Services
264,358	10.04	Hypertension
83,592	3.17	Diabetes
42,605	1.62	Infective nasopharyngitis NOS
24,211	0.92	Hypothyroidism
24,097	0.91	Headache
23,210	0.88	Urinary tract infection
21,122	0.80	Low back pain
19,998	0.76	Hyperlipidemia
870,245	33.04	Other’s

To further characterize the health status of TM users, we analyzed the presence of chronic conditions registered prior to their first TM contact. Table 3 summarizes the top 10 Chronic comorbidities in the TM users and the overall insured population. Among TM users, hypertension was the most prevalent pre-existing condition (15.0%), followed by asthma (3.9%), hyperlipidemia (3.3%), and type 2 diabetes (3.1%). Among the general insured population, the most prevalent pre-existing condition was hypertension (11.9%), hyperlipidemia (9.0%), asthma (4.2%), and type 2 diabetes (3.8%).

**Table 3:** Chronic conditions among telemedicine users vs. health plan population as a whole.

Characteristics	Telemedicine users (*N =* 2,633,564)	Health plan population (*N =* 10,016,931)
Preexisting conditions (%)		
Hypertension	396,614 (15.0)	608,016 (11.9)
Hyperlipidemia	85,590 (3.3)	458,295 (9.0)
Asthma	104,815 (3.9)	217,728 (4.2)
Diabetes	40,030 (1.5)	191,337 (3.8)
Chronic Kidney Disease	4,213 (0.2)	81,711 (1.6)
Cancer^	9,480 (0.4)	66,993 (1.3)
Chronic Obstructive Pulmonary Disease (COPD)	6,320 (0.2)	62,933 (1.24)
Autoimmune Diseases	12,904 (0.5)	50,245 (0.9)
HIV	8,427 (0.3)	21,823 (0.4)
Palliative care program*	1,053 (0.04)	4,567 (0.09)

^^^Limited to adult cancers. ^*^In Colombia those who are enrolled in palliative care must have a terminal chronic condition.

TM users stratified by income level. In the subsidized group (predominantly unemployed), 70.0% of users were female whereas 56.1% were in the high-income group (Table 4). Approximately 52% of TM users aged 19–45 years were in the subsidized group whereas 55.3% were in the high-income group. Most TM users were mestizo ethnicity in both the subsidized (74.2%) and high-income groups. Finally, 86.8% of TM users who were in the subsidized group had occupational degree and 80.0% who had occupational degree were in the higher-income group. In terms of number of preexisting conditions, a higher proportion of TM users had zero followed by 1 and 2 across all income level groups (Table 5).

**Table 4:** Sociodemographic characteristics by income level among the TM population.

Characteristics	Subsidized (*N =* 69,502)	Low income (*N =* 1,711,007)	Middle income (*N =* 605,574)	High income (*N =* 247,481)
Female sex – no (%)	48,636 (70.0)	1,092,812 (63.9)	374,042 (61.8)	138,912 (56.1)
Age group – no (%)				
0–5 yr	2,927 (4.2)	38,065 (2.2)	10,429 (1.7)	4,214 (1.7)
6–10 yr	1,679 (2.4)	26,679 (1.6)	8,046 (1.3)	3,508 (1.4)
11–18 yr	3,297 (4.7)	60,893 (3.6)	15,958 (2.6)	7,649 (3.1)
19–45 yr	35,784 (51.5)	929,194 (54.3)	355,682 (58.7)	136,888 (55.3)
45–60 yr	16,421 (23.6)	330,420 (19.3)	104,900 (17.3)	53,713 (21.7)
61–70 yr	6,269 (9.0)	173,576 (10.1)	58,303 (9.6)	21,801 (8.8)
>70 yr	3,125 (4.5)	152,180 (8.9)	52,256 (8.6)	19,708(7.9)
Race – no (%)^^				
Black	1,598 (5.1)	21,749 (2.9)	5,771 (2.3)	1,651 (2.1)
White	6,205 (19.9)	185,818 (25.5)	67,018 (27.4)	23,129 (29.7)
Mulatto	172 (0.55)	2,638 (0.3)	659 (0.2)	150 (0.1)
Indigenous	18 (0.06)	489 (0.07)	86 (0.04)	38 (0.05)
Mestizo	23,053 (74.2)	515,538 (70.8)	170,506 (69.7)	52,780 (67.8)
Zambo	43 (0.14)	1,122 (0.15)	259 (0.11)	100 (0.13)
Highest Education Level – no (%)				
Primary School	963 (4.0)	28,882 (4.7)	7,720 (3.6)	2,272 (2.3)
High School	2,088 (8.7)	89,556 (15)	29,293 (14)	8,893 (9.0)
Occupational degree^a^	20,885 (86.8)	486,556 (80)	169,399 (79)	79,076 (80)
Undergraduate	93 (0.39)	6,941 (1.1)	6,263 (3)	5,891 (5.9)
Graduate^b^	30(0.12)	1,588 (0.26)	1,805 (0.84)	3,829(3.83)

^^^^Races in Latino America: Mestizo: Indigenous + European, Mulato: African + European, Zambo: Black + Indigenous. ^a^Occupations such as electricians and chefs can achieve specific post-high school occupational training.. ^b^Graduate programs include Specialization, Master’s, PhD and Postdoc.

**Table 5:** Number of preexisting conditions of telemedicine users by income level.

Characteristics	Subsidized (*N =* 69,502)	Low income (*N =* 1,711,007)	Middle income (*N =* 605,574)	High income (*N =* 247,481)
# Previous Diseases – no (%)				
0	31,662 (45.6)	743,930 (43.5)	288,794 (47.7)	138,807 (56.1)
1	14,253 (20.5)	361,890 (21.2)	125,004 (20.6)	43,172 (17.4)
2	9,752 (14.0)	236,659(13.8)	78,738 (13.0)	27,837 (11.2)
3	7,624 (11.0)	189,279 (11.1)	60,091 (9.9)	20,980 (8.5)
4	3,883 (5.6)	104,711 (6.1)	32,317(5.3)	10,660 (4.3)
5	1,546 (2.2)	46,154 (2.7)	13,007 (2.1)	4,025 (1.6)
6	574 (0.8)	17,957 (1.0)	4,916 (0.8)	1,428 (0.6)
7	173 (0.2)	6,751 (0.4)	1,960 (0.3)	408 (0.2)
8	21 (0.0)	2,584 (0.2)	549 (0.1)	138 (0.1)
9	10 (0.0)	776 (0.0)	158 (0.0)	19 (0.0)
10	4 (0.0)	211 (0.0)	26 (0.0)	6 (0.0)
11	0 (0.0)	34 (0.0)	8 (0.0)	0 (0.0)
12	0 (0.0)	72 (0.0)	6 (0.0)	0 (0.0)

### 3.3 Geographical distribution of telemedicine use

For the graphical analysis, we used ArcGIS Pro as the GIS mapping software to plot the latitude and longitude coordinates. A heat map was generated according to the distribution of users. Red dots represent concentrations of more than 100,000 patients, yellow dots represent 50,000–100,000 patients, and green dots represent fewer than 50,000 users.

In addition, we created a 3D map of Primary Care Units (PCUs) across Colombia, reflecting the places affiliated with users at the time of their telemedicine consultation. To protect confidentiality, we did not use the exact addresses provided during medical consultations. A total of 108 PCUs across the country were mapped using a geographic information system (GIS). The greatest density of TM use was concentrated in urban areas, particularly in the departments of Antioquia, Bogotá D. C., Valle del Cauca (Cali), and Atlántico (Barranquilla).

**Figure 1:**
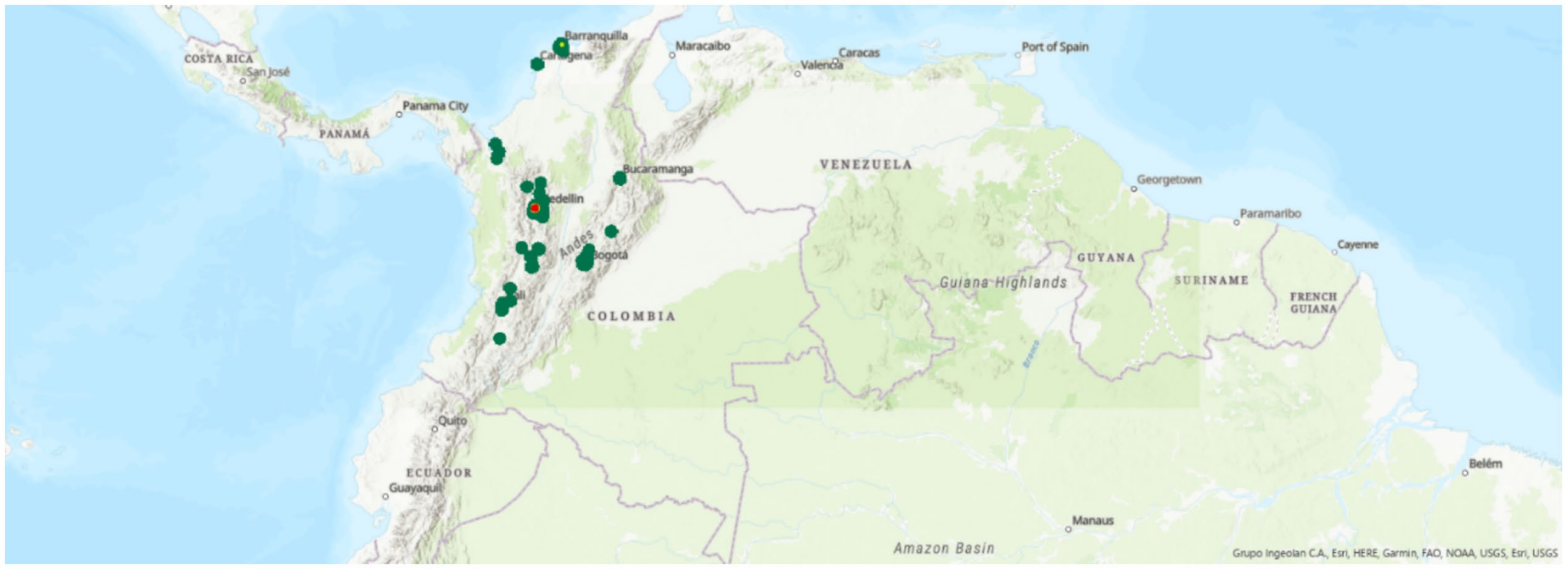
A GIS map displaying the geographical location of primary care units for the telemedicine users between March 2020 and June 2021. A total of 108 primary care units distributed between the center and north of Colombia. 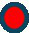 >100.000 High concentration of TM users. 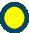 100.000–50.000 Middle concentration of TM users. 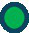 < 50.000 Less concentration of TM users.

**Figure 2:**
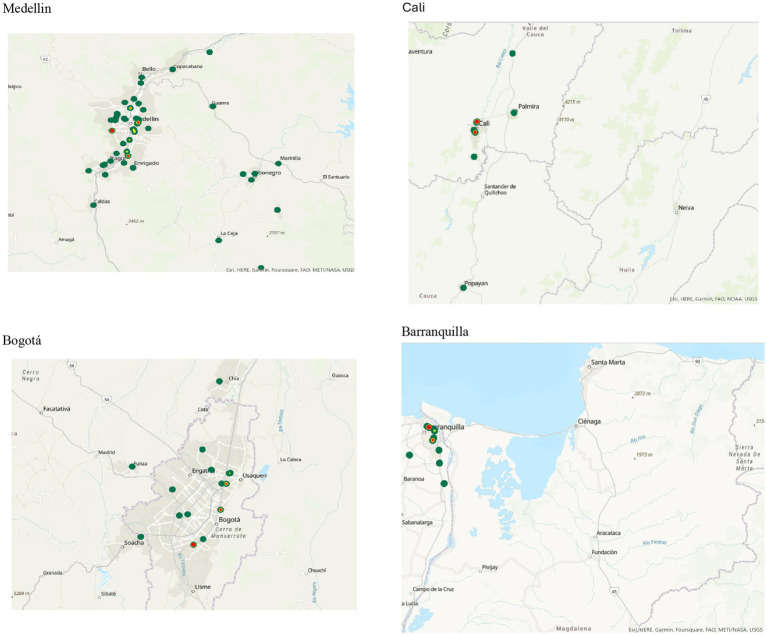
A GIS map showing the location of primary care units in the main cities of Colombia, including Medellin, Bogotá, Cali, and Barranquilla. (A) Medellin. (B) Bogotá. (C) Cali. (D) Barranquilla.

Figure 1 presents a national heat map illustrating the distribution of TM consultations across Colombia. Figure 2 illustrates the four main cities of Colombia—Medellín, Cali, Bogotá, and Barranquilla—and specifically highlights the areas of concentration of the patients’ Primary Care Units (PCUs), allowing the reader to visualize their geographic distribution in the urban, semiurban and rural areas.

## 4 Discussion

The aim of this study was to describe the sociodemographic characteristics and health conditions of telemedicine (TM) users within a large Colombian insurance provider during the COVID-19 pandemic. Our principal findings show that over 2.6 million insured individuals used telemedicine services, spanning diverse demographic groups and health conditions (8–10). The adoption of telemedicine occurred not only among individuals with chronic conditions, such as hypertension and diabetes, but also in a wide range of non-chronic conditions. Usage extended to both pediatric and older adult populations, indicating a systematic shift in the delivery of care that transcended age, gender and socioeconomic status.

One of the most significant findings is that a higher proportion of individuals from low-income and subsidized groups were primary users of telemedicine. This challenges the pervasive notion that digital health may inherently widen inequalities due to disparities in internet access or digital literacy (14, 15). In our context, telemedicine appeared to function as a mechanism of health equity, allowing vulnerable populations to access timely care without the barriers of transportation costs, time off work, or exposure to infections (14). This is consistent with findings in other settings, such as in the United States, where Cantor et al. (2021) showed that telehealth expansion during the pandemic helped maintain access across sociodemographic groups (16). However, it contrasts with findings in low-resource settings like Ghana and Nigeria, where Dodoo et al. (17) described persistent inequalities in telemedicine access, emphasizing that context-specific infrastructure and governance play a decisive role in shaping digital health equity.

Women accounted for nearly two-thirds of all TM users. This may reflect increased healthcare-seeking behavior among women but could also point to their central role in family health management, particularly during public health emergencies (18, 19). These findings mirror regional and global patterns, where women are more likely to access primary care and chronic disease services, and may be disproportionately responsible for coordinating care for children and older relatives. From a geographical standpoint, telemedicine services were delivered across nearly all departments of Colombia, even though the insurer does not operate in every region. The strategic deployment of primary care units (PCUs) as nodes for virtual care made it possible to overcome some of the limitations imposed by the country’s persistent digital divide (20). The Economic Commission for Latin America and the Caribbean (CEPAL, 2020) has emphasized that such digital gaps remain one of the most pressing structural challenges to inclusive telehealth in the region (20). Nevertheless, the present study shows that even in a middle-income country with unequal connectivity, a rapid and massive adoption of telemedicine was possible, highlighting the latent capacity for digital transformation within health systems when aligned with urgent public health needs (21, 22).

Importantly, the structure and timing of this study offer unique insights. Unlike most reports from Colombia during the pandemic, which focused on specific hospitals, cities, or disease categories, our analysis captures the full spectrum of patients utilizing telemedicine in a large insurance network. It reveals not only who used telemedicine, but also how usage patterns varied by sociodemographic and clinical factors—critical information for scaling digital health solutions in a post-pandemic landscape. The inclusion of diagnostic categories further enriches our understanding of the types of conditions managed virtually, ranging from respiratory symptoms and acute infections to endocrine and mental health conditions. These results also provide empirical support for the conceptualization of telemedicine not as a temporary substitute during crisis, but as a viable and scalable modality for sustained healthcare delivery (23, 24). The rapid uptake of virtual consultations by both providers and patients underscores a level of digital readiness in the Colombian health system that had not previously been quantified. Moreover, the shift was accomplished without significant exclusions along income or age lines, suggesting a window of opportunity for institutionalizing telemedicine as part of the routine healthcare model.

For health professionals, these findings highlight the need for strengthened training in telemedicine competencies to ensure high-quality virtual care. For policymakers, they emphasize the importance of sustained investments in digital infrastructure, equitable findings models, and comprehensive regulatory frameworks to guarantee continuity, quality, and data protection. For researchers, future work should assess patient outcomes, satisfaction, and long-term health impacts; explore provider perspectives and barriers to adoption; and conduct longitudinal and comparative analyses to evaluate the sustainability and equity of telemedicine services (20). Taken together, the Colombian experience demonstrates that, beyond its role in public health emergencies, telemedicine has the potential to address persistent challenges in healthcare delivery – such as geographic inequities, provider shortages, continuity of care for chronic conditions, and access for marginalized populations. It should no longer be viewed merely as an emergency response, but as a strategic component of resilient, patient-centered health systems capable of adapting to the evolving needs of diverse populations.

## 5 Limitations

This study offers valuable insights into telemedicine use in Colombia; however, some limitations should be acknowledged. While the dataset encompasses a large and diverse sample of over 2.6 million individuals, it represents enrollees from a single health insurance provider, which may not fully capture the heterogeneity of the national population. Nonetheless, this insurer covers a broad geographic and demographic spectrum, offering a meaningful lens into real-world implementation. Additionally, to protect patient confidentiality, geographic information was limited to primary care unit assignments, restricting detailed analysis of urban–rural disparities. Although we did not include measures of digital literacy, internet access, or user experience—key aspects of digital equity—our findings still provide a foundational understanding of sociodemographic access patterns. Finally, while disaggregation of some variables was limited due to privacy considerations, the overall trends observed offer a robust starting point for future research and policy development in digital health.

### 5.1 Implications for policy and practice

The findings have clear implications for public health strategy and health system planning in Colombia and similar LMICs. First, they confirm that telemedicine can be an equity-promoting intervention when deployed at scale and integrated into existing care networks, especially primary care. To maintain and expand this success, investments must be made in broadband connectivity, especially in underserved regions, and in the digital capacity of primary care units and health professionals. Policies that ensure telemedicine reimbursement parity, promote interoperability between platforms, and establish quality-of-care benchmarks will be critical for long-term sustainability. Furthermore, national telehealth strategies must prioritize digital inclusion by addressing structural barriers such as internet affordability and technological accessibility. Digital health literacy campaigns and community-based education efforts can help close usage gaps and empower patients to take an active role in their healthcare. Lessons from this study can guide both the refinement of telemedicine infrastructure and the design of hybrid care models that combine virtual and in-person services according to clinical appropriateness and patient preference.

## 6 Conclusion

This study provides population-level evidence that telemedicine was central to preserving healthcare access in Colombia during the COVID-19 pandemic. Its widespread adoption across age, gender, and socioeconomic groups underscores its role as a mechanism for advancing health equity rather than serving solely as a temporary substitute. The Colombian experience demonstrates the country’s capacity for digital health innovation and offers a scalable reference for other LMICs seeking to integrate telemedicine into routine care. To secure lasting transformation, sustained political commitment, strategic infrastructure investment, and equity-oriented policies will be essential.

## Data Availability

The raw data supporting the conclusions of this article will be made available by the authors, without undue reservation.
